# Four‐Chamber Deformation Remodeling and Atrial Fibrillation After Septal Myectomy for Obstructive Hypertrophic Cardiomyopathy

**DOI:** 10.1111/echo.70567

**Published:** 2026-07-28

**Authors:** Olga N. Kislitsina, Lubna Choudhury, Kifah Hussain, James D. Thomas, Seokyung An, Abigail S. Baldridge, Daniel J. Romary, Beth Whippo, James L. Cox, Patrick M. McCarthy, Douglas R. Johnston

**Affiliations:** ^1^ Division of Cardiac Surgery Feinberg School of Medicine and the Bluhm Cardiovascular Institute Northwestern University Chicago Illinois USA; ^2^ Division of Cardiology Feinberg School of Medicine and the Bluhm Cardiovascular Institute Northwestern University Chicago Illinois USA

**Keywords:** 4‐chamber cardiac strain analysis, echocardiography, hypertrophic obstructive cardiomyopathy, NYHA functional class

## Abstract

**Introduction:**

Septal myectomy treats hypertrophic obstructive cardiomyopathy (HOCM), but its effect on myocardial deformation across cardiac chambers is incompletely defined. We evaluated chamber‐specific strain remodeling 1 year after septal myectomy and examined whether atrial strain was associated with atrial fibrillation (AF) during follow‐up.

**Methods:**

We retrospectively studied 187 consecutive patients undergoing surgical septal myectomy for HOCM. Conventional echocardiography and speckle‐tracking strain analysis were used to assess left ventricular (LV), right ventricular (RV), left atrial (LA), and right atrial (RA) deformation preoperatively and at 1‐year follow‐up. Paired analyses compared preoperative and postoperative strain values. Multivariable logistic regression evaluated associations between baseline strain measures, AF, and all‐cause mortality.

**Results:**

Among 187 patients, 85 had analyzable 1‐year strain studies. At 1 year, LA contractile strain (CS) improved from 10.4±5.9% to 13.6±7.1% (*p* = 0.005), whereas LA reservoir strain (RS) did not significantly change. In contrast, RA‐RS decreased from 36.0±10.6% to 28.0±11.6% (*p* = 0.001), RV free‐wall strain worsened from −26.3±7.0% to −21.1±6.8% (*p* = 0.001), and RV global longitudinal strain (GLS) worsened from −21.3±5.1% to −17.3±5.5% (*p* = 0.001). LV‐GLS was unchanged. Higher LA‐CS was associated with lower odds of AF after adjustment (aOR 0.92, 95% CI 0.85–0.99; *p* = 0.018), as was higher LA‐RS (aOR 0.95, 95% CI 0.91–0.99; *p* = 0.035). LA‐RS was associated with mortality in unadjusted analysis but not after multivariable adjustment.

**Conclusions:**

Septal myectomy is followed by nonuniform, chamber‐specific deformation remodeling. LA‐CS improves, LA‐RS and CS identify patients at higher AF risk, LV‐GLS remains unchanged, and right‐sided longitudinal deformation worsens at 1 year. Four‐chamber strain analysis may augment post‐myectomy surveillance.

## Graphical abstract

After septal myectomy for hypertrophic obstructive cardiomyopathy, the four chambers of the heart experience unique remodeling patterns that can be visualized with speckle‐tracking strain analysis on echocardiography. This study characterizes those changes and links them to clinical outcomes such as post‐operative atrial fibrillation risk, thereby augmenting post‐myectomy surveillance.

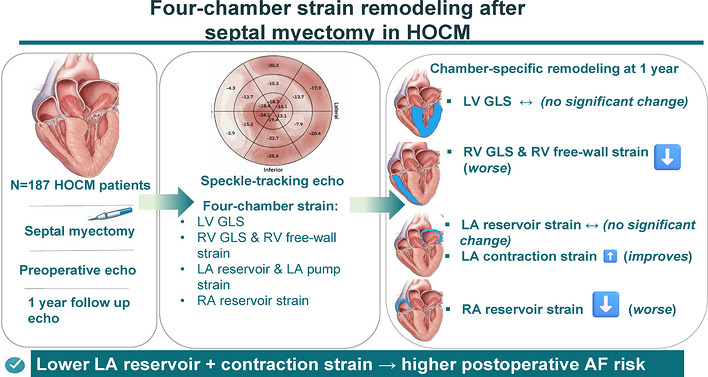

**Abbreviations**
AF: atrial fibrillation
CPB: cardiopulmonary bypass
FAC: fractional area change
HF: heart failure
HOCM: hypertrophic obstructive cardiomyopathy
LA: left atrial
LV‐GLS: left ventricular global longitudinal strain
LVOT: left ventricular outflow tract obstruction,
RA: right atrial
RV: right ventricle
RV FWS: right ventricle free‐wall strain
SCD: sudden cardiac death

## 1 Introduction

Hypertrophic obstructive cardiomyopathy (HOCM) is a disorder characterized by left ventricular (LV) hypertrophy, dynamic LV outflow tract (LVOT) obstruction, systolic anterior motion (SAM) of the mitral valve, and mitral regurgitation [1, 2]. Septal myectomy relieves the LVOT gradient and improves symptoms and long‐term survival [3, 4]. However, post‐myectomy risks of major adverse cardiac events persist, including atrial fibrillation (AF), heart failure (HF), and sudden cardiac death (SCD) [5]. These risks underscore the need to identify better predictors of long‐term prognosis that extend beyond the relief of LVOT obstruction.

HOCM manifests histologically as local septal myocyte enlargement, pleomorphism, and myocyte disarray [6]. In addition, small‐vessel disease and interstitial fibrosis cause collagen deposition between myocytes resulting in LV diastolic dysfunction and a pro‐arrhythmic substrate [7, 8]. Diffuse areas of regional contractile dysfunction usually go undetected by standard echocardiographic parameters, but speckle‐tracking echocardiography analysis provides a means of detecting and quantitating regional and global myocardial strain [9, 10]. This more sensitive imaging technique reveals that despite preserved LVEF, LV global longitudinal strain (LV‐GLS) is frequently impaired in patients with HOCM and is associated with adverse outcomes [11]. Prospective data indicate that LV‐GLS may remain unchanged after septal myectomy even as clinical status and diastolic indices improve, suggesting a potential dissociation between obstruction relief and subsequent myocardial remodeling. Unfortunately, most of these prior septal myectomy imaging studies have focused on LV remodeling and LVOT gradients, or on LV and left atrial (LA) strain alone.

It is well established that reduced LA reservoir and booster strain are markers of atrial myopathy and are associated with AF in patients with HCM and HOCM [12]. This is clinically important because relief of LVOT obstruction does not necessarily reverse the atrial substrate that contributes to post‐myectomy exercise intolerance and AF [13, 14]. In contrast, far less is known about right atrial (RA) and right ventricular (RV) deformation after septal myectomy. This gap in knowledge is relevant for two reasons. First, HOCM is not purely a left‐sided outflow tract disease, and right‐sided myocardial involvement may contribute to persistent symptoms after successful relief of obstruction. Second, open cardiac surgery with cardiopulmonary bypass, cardioplegic arrest, and pericardiotomy can depress RV longitudinal performance [15, 16]. Therefore, we measured strain in all four cardiac chambers before and 1 year after septal myectomy for HOCM to determine whether postoperative deformation remodeling is uniform or chamber‐specific. We also examined whether atrial strain, as a quantitative marker of atrial myopathy, was associated with postoperative AF and functional recovery.

## 2 Methods

### 2.1 Study Design and Study Population

We performed a retrospective cohort study of consecutive patients with HOCM who underwent surgical septal myectomy at Northwestern Memorial Hospital between January 2005 and October 2024. The full surgical cohort included 187 patients and was used to describe baseline clinical, echocardiographic, operative, and postoperative outcomes. Patients were included because septal myectomy was performed for obstructive HCM; concomitant procedures were performed when clinically indicated but did not define study eligibility. A subset of patients had preoperative and approximately 1‐year postoperative transthoracic echocardiograms of sufficient quality for four‐chamber speckle‐tracking strain analysis. This paired strain cohort included 85 patients, although analyzable sample size varied by chamber and strain parameter because of image quality and view availability.

Clinical, operative, and follow‐up data were obtained from the Cardiovascular Research Database at the Clinical Trial Unit of the Bluhm Cardiovascular Institute, Northwestern Memorial Hospital, and supplemented by review of the electronic medical record when necessary. The study was approved by the Northwestern University Institutional Review Board (STU00012288), and written informed consent was obtained for longitudinal database participation.

### 2.2 Transthoracic Echocardiography Protocol and Strain Imaging Analysis

Transthoracic echocardiography was performed according to American Society of Echocardiography recommendations. Conventional echocardiographic measures included LV size and systolic function, interventricular septal thickness, LA size and volume, Doppler‐derived indices of diastolic function, LVOT gradients, mitral regurgitation severity, and right‐sided measures including tricuspid annular plane systolic excursion, tissue Doppler velocities, and estimated RV systolic pressure when available. The LVOT gradients reported in the baseline echocardiographic table represent resting transthoracic Doppler measurements; provoked or exercise gradients were considered in surgical decision‐making when available but were not uniformly captured as standardized analytic variables.

Digitally archived echocardiographic images were analyzed offline using TomTec‐Arena, Philips Ultrasound Workspace version 51.07, to derive longitudinal strain measurements. Strain was measured in the left ventricle, right ventricle, left atrium, and right atrium. LV global longitudinal strain was derived from standard apical views using a multi‐segment model. RV strain was measured from the best available RV‐focused or apical four‐chamber view and included RV free‐wall strain and RV global longitudinal strain. LA and RA strain were measured from apical four‐chamber views and included reservoir strain and contractile, or pump, strain [17]. Conduit strain was not included in the present analysis.

For postoperative strain analysis, follow‐up studies were required to have adequate endocardial border definition, minimal foreshortening, stable electrocardiographic gating, and a frame rate of at least 40 frames/s. Ventricular longitudinal strain is reported as a negative percentage, with more negative values reflecting greater systolic shortening. Atrial strain is reported as a positive percentage, with higher values reflecting better reservoir or contractile deformation [18]. LA and RA contractile strain (CS) were measured only when atrial contraction could be identified; patients in AF at the time of image acquisition were excluded from CS analysis for that examination. Readers were blinded to clinical outcomes during offline strain analysis.

### 2.3 Surgical Management

Septal myectomy was performed using a standard transaortic, Morrow‐type surgical approach. Resection was directed at the hypertrophied basal septum and extended distally as needed based on preoperative imaging, intraoperative anatomy, and operative assessment of the LVOT. Intraoperative transesophageal echocardiography was used to assess residual obstruction and mitral regurgitation. Concomitant procedures were recorded, including mitral valve repair or replacement, coronary artery bypass grafting, tricuspid valve surgery, and surgical ablation for AF. Operative variables included cardiopulmonary bypass time, aortic cross‐clamp time, blood product use, and need for concomitant procedures when available. Quantitative myectomy length and width were not systematically recorded across the study period and therefore could not be included as analytic variables.

### 2.4 Outcomes

The primary imaging endpoint was the change in four‐chamber strain from the preoperative echocardiogram to the approximately 1‐year postoperative echocardiogram among patients with paired analyzable studies.

The primary clinical endpoint for association analyses was AF during follow‐up. AF was defined as ECG‐documented AF, a clinical diagnosis of AF recorded in the electronic medical record, or postoperative AF documented after septal myectomy. Because prior AF may influence postoperative rhythm outcomes, history of AF was included as an adjustment variable in multivariable models. In addition, a sensitivity analysis was performed after excluding patients with preoperative AF to evaluate predictors of new‐onset AF during follow‐up. Secondary exploratory outcomes included all‐cause mortality, heart failure hospitalization, and ventricular tachycardia. Sudden cardiac death was not analyzed separately because of the limited number of events.

### 2.5 Statistical Analysis

Continuous variables are summarized as mean ± SD or median (Q1, Q3), as appropriate, and categorical variables as counts and percentages. Inter‐group comparisons used unequal‐variance two‐sample t tests or Wilcoxon rank‐sum tests for continuous variables and chi‐square or Fisher exact tests for categorical variables. Paired comparisons of preoperative versus 1‐year strain values used paired statistical tests as appropriate.

Associations between strain and outcomes were evaluated using logistic regression, reporting odds ratios per 1% difference in strain. Multivariable models adjusted for clinically relevant covariates available in the dataset, including age, sex, diabetes mellitus, hypertension, CHA_2_DS_2_‐VASc score, history of AF, history of myocardial infarction, prior cardiovascular intervention, and preoperative SAM status. For the new‐onset AF sensitivity analysis, patients with preoperative AF were excluded, and history of AF was not included as a covariate. Receiver operating characteristic (ROC) analyses were used to evaluate discrimination and explore candidate strain cut points for selected outcomes. Kaplan–Meier methods and log‐rank tests were used for time‐to‐event comparisons by SAM status. A two‐sided alpha of 0.05 was used.

## 3 Results

### 3.1 Study Population and Baseline Clinical Status

Mean age was 56.9 ± 15.6 years, 57.8% had hypertension, and 15.0% had diabetes mellitus (Table 1). A prior history of AF was present in 19.3%. Preoperatively, 38% of patients were in NYHA class III HF and 5.9% were in class IV HF.

**TABLE 1:** Baseline clinical and operative characteristics.

Characteristic	Entire cohort (*N* = 187)
**Baseline clinical characteristics**	
Age, years	56.9 ± 15.6
Body surface area, m^2^	2.0 ± 0.3
Body mass index, kg/m^2^	29.3 ± 6.0
CHA_2_DS_2_‐VASc score	2 (1, 3)
Creatinine, mg/dL	1.0 (0.8, 1.1)
Diabetes mellitus, No. (%)	28 (15.0%)
Hypertension, No. (%)	108 (57.8%)
Prior myocardial infarction, No. (%)	7 (3.8%)
Prior cardiovascular intervention, No. (%)	39 (20.9%)
Congestive heart failure history, No. (%)	56 (29.9%)
Atrial fibrillation history, No. (%)	36 (19.3%)
Preoperative systolic anterior motion, No. (%)	157 (84.0%)
**NYHA functional class**	
Class I	34 (18.2%)
Class II	44 (23.5%)
Class III	71 (38.0%)
Class IV	11 (5.9%)
Not documented	27 (14.4%)
**Operative characteristics**	
Elective operation, No. (%)	178 (95.2%)
Urgent operation, No. (%)	9 (4.8%)
Intraoperative blood products, No. (%)	87 (46.5%)
Mitral valve surgery, No. (%)	132 (70.6%)
Coronary artery bypass grafting, No. (%)	19 (10.2%)
Surgical AF ablation, No. (%)	31 (16.6%)
Tricuspid valve surgery, No. (%)	2 (1.1%)
ASD/PFO repair, No. (%)	5 (2.7%)

*Notes*: Values Are Mean ± SD, Median (Q1, Q3).

Abbreviations: AF, atrial fibrillation; ASD, atrial septal defect; NYHA, New York Heart Association; PFO, patent foramen ovale.

All patients underwent septal myectomy for obstructive HCM. Concomitant procedures included mitral valve surgery or mitral apparatus intervention in 70.6%, CABG in 10.2%, and AF ablation in 16.6%. The high rate of mitral intervention reflects SAM‐related mitral regurgitation or mitral apparatus abnormalities in a tertiary HOCM referral cohort. Operative mortality was 2.7%. Pre‐discharge permanent pacemaker implantation was required in 9.6% (Table S1).

### 3.2 Preoperative Conventional Echocardiography and Strain Parameters

**FIGURE 1:**
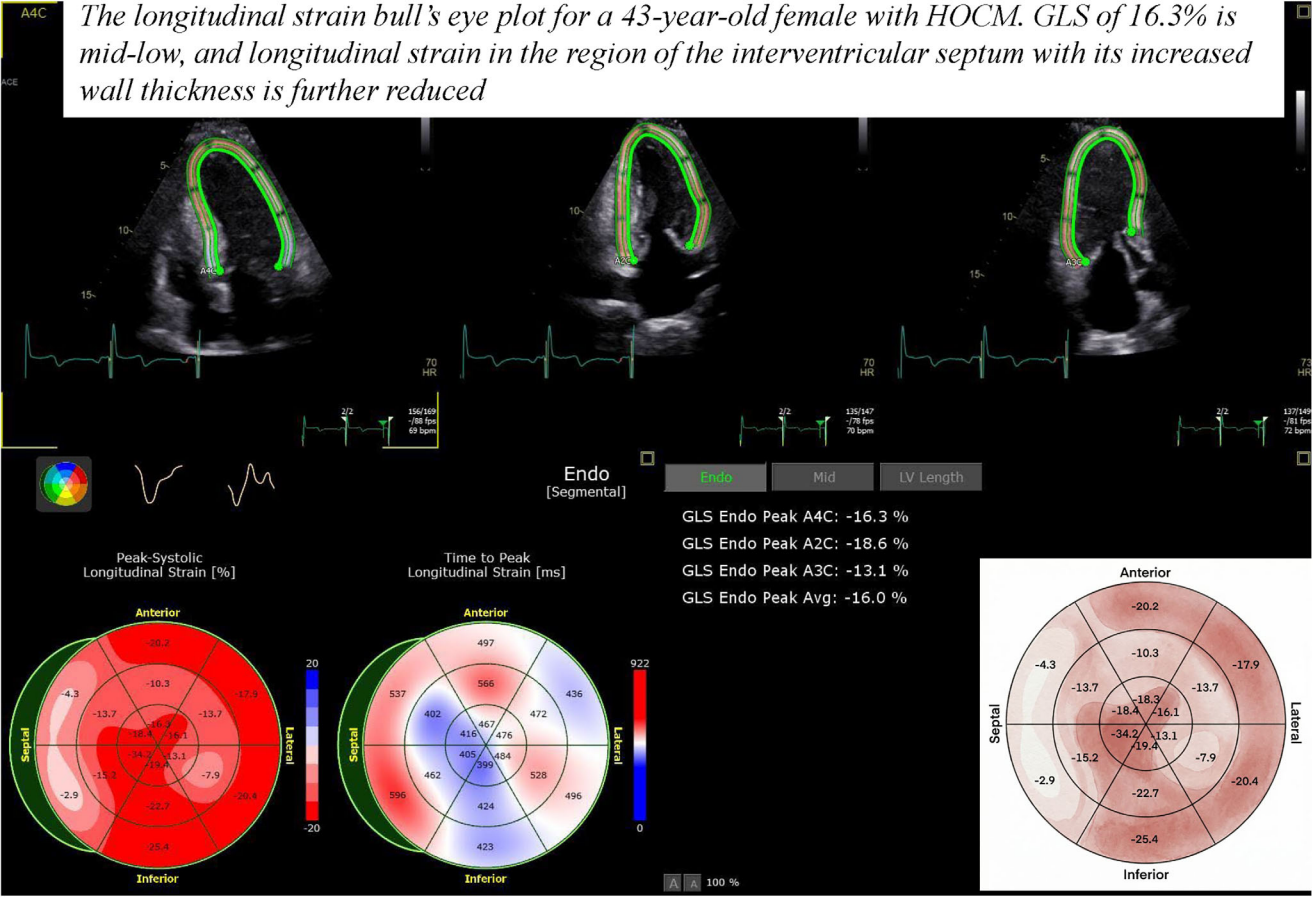
Representative LV longitudinal strain in HOCM. Apical 4‐, 2‐, and 3‐chamber speckle‐tracking views with corresponding bullseye plot demonstrate heterogeneous peak systolic longitudinal strain, with relatively reduced septal strain compared with other segments, despite only mildly reduced global longitudinal strain.

Preoperative LV systolic function was generally normal (Table 2). Resting LVOT obstruction was present, with a median LVOT peak gradient of 36.0 mmHg and median LVOT mean gradient of 6.0 mmHg. Median LV‐GLS was −18.6%. Although LV‐GLS was only mildly abnormal, LV strain maps frequently demonstrated localized regional dysfunction (Figure 1). RV deformation was similarly reduced, with RV‐GLS of −21.1% and RV free‐wall strain of −26.7%. LA enlargement was prominent and LA function was impaired, with LA reservoir strain of 25.1% and LA contraction strain of 10.0%. RA function was also abnormal, with RA reservoir strain of 38.2% and RA contraction strain of 14.5%.

**TABLE 2:** Conventional echocardiographic and four‐chamber strain characteristics.

Parameter	Value
**Preoperative conventional echocardiography**	
Interventricular septal thickness, IVSd, cm	1.8 (1.5, 2.1)
Left atrial diameter, cm	4.2 (3.9, 4.6)
Left atrial volume, mL	93.8 (71.4, 117.3)
LV internal diameter, diastole, cm	4.2 (3.7, 4.6)
LV internal diameter, systole, cm	2.4 (2.2, 2.7)
LV end‐diastolic volume, mL	93.0 (72.3, 108.8)
LV end‐systolic volume, mL	28.7 (21.9, 37.1)
LV ejection fraction, %	66.2 (61.6, 72.7)
LVOT mean gradient, mmHg	6.0 (3.6, 9.1)
LVOT peak gradient, mmHg	36.0 (15.0, 72.0)
E/e′ ratio	14.3 (10.0, 22.4)
RV systolic pressure, mmHg	32.3 (26.0, 41.4)
TAPSE, cm	2.3 (1.7, 2.6)
Tricuspid regurgitation peak gradient, mmHg	27.6 (22.1, 34.9)
**Preoperative four‐chamber strain**	
LV global longitudinal strain, %	−18.6 (−20.3, −16.5)
RV global longitudinal strain, %	−21.1 (−25.0, −17.7)
RV free‐wall longitudinal strain, %	−26.7 (−31.1, −22.2)
LA reservoir strain, %	25.1 (18.5, 32.0)
LA contractile strain, %	10.0 (6.0, 14.3)
RA reservoir strain, %	38.2 (30.6, 43.0)
RA contractile strain, %	14.5 (10.0, 19.0)
**One‐year follow‐up four‐chamber strain**	
LV global longitudinal strain, %	−17.2 (−20.2, −13.4)
RV global longitudinal strain, %	−17.2 (−21.2, −14.2)
RV free‐wall longitudinal strain, %	−21.8 (−25.7, −17.3)
LA reservoir strain, %	24.6 (16.6, 34.3)
LA contractile strain, %	12.7 (7.8, 18.9)
RA reservoir strain, %	29.8 (19.1, 35.7)
RA contractile strain, %	13.0 (8.3, 19.2

*Notes*: Values Are Median (Q1, Q3) Unless Otherwise Specified.

Abbreviations: LA, left atrial; LV, left ventricular; LVOT, left ventricular outflow tract; RA, right atrial; RV, right ventricular; TAPSE, tricuspid annular plane systolic excursion. Ventricular strain values are expressed as negative percentages. LVOT gradients are resting transthoracic Doppler measurements.

### 3.3 One‐Year Follow‐up Imaging

One‐year follow‐up showed LV‐GLS of −17.2%, RV‐GLS of −17.2%, RV‐FWS of −21.8%, LA reservoir strain of 24.6%, LA contraction strain of 12.7%, RA reservoir strain of 29.8%, and RA contraction strain of 13.0%. Paired comparisons of standard echocardiographic metrics are shown in Table S2. LVOT peak gradient decreased from 50.6 ± 46.7 mmHg preoperatively to 10.4 ± 14.9 mmHg at follow‐up (*p* = 0.001). Septal thickness decreased from 1.8 ± 0.4 cm preoperatively to 1.4 ± 0.4 cm at follow‐up (*p* = 0.004). LA volume decreased from 96.9 ± 32.6 mL preoperatively to 74.2 ± 26.9 mL following surgery (*p* = 0.006). RV systolic pressure decreased from 36.2 ± 15.9 mmHg preoperatively to 28.1 ± 6.5 mmHg at 1 year postoperatively (*p* = 0.030).

### 3.4 Paired Four‐Chamber Strain Changes After Myectomy

Paired pre‐ and postop comparisons are summarized in Tables S2 and S3 and demonstrate non‐uniform remodeling across all four chambers. LA contractile function improved during follow‐up (*p* = 0.005) but LA reservoir strain did not change (*p* = 0.21). RA CS remained unchanged (*p* = 0.62) but RA reservoir strain decreased significantly (*p* = 0.001). Both RV‐GLS and RV‐FWS worsened during follow‐up. However, LV‐GLS did not change significantly from baseline to follow‐up (*p* = 0.17).

### 3.5 Associations Between Strain Parameters and Clinical Outcomes

In unadjusted analysis, higher LA reservoir strain was associated with lower odds of mortality (OR 0.95 per 1% increase; 95% CI, 0.90–0.99; *p* = 0.015, but this association was not significant after multivariable adjustment (adjusted OR 0.98; 95% CI, 0.93–1.03; *p* = 0.41). No adjusted LV, RV or RA strain parameter was independently associated with mortality (Table 3).

**TABLE 3:** Exploratory association between baseline four‐chamber strain and all‐cause mortality.

Strain parameter	N	Unadjusted OR (95% CI)	*p* value	Adjusted OR (95% CI)	*p* value
LA contractile strain, %	164	0.96 (0.90–1.04)	0.32	0.98 (0.90–1.06)	0.59
LA reservoir strain, %	169	0.95 (0.90–0.99)	0.015	0.98 (0.93–1.03)	0.41
LV global longitudinal strain, %	169	1.01 (0.92–1.10)	0.91	0.94 (0.83–1.08)	0.41
RA contractile strain, %	151	1.04 (0.97–1.10)	0.26	1.05 (0.97–1.13)	0.25
RA reservoir strain, %	161	0.99 (0.95–1.03)	0.60	1.01 (0.97–1.06)	0.56
RV free‐wall longitudinal strain, %	168	1.00 (0.95–1.06)	0.98	1.00 (0.93–1.07)	0.94
RV global longitudinal strain, %	168	1.02 (0.94–1.10)	0.63	1.02 (0.93–1.11)	0.73

*Note*: ORs are reported per 1% increase in strain. Multivariable models were adjusted for age, sex, diabetes mellitus, hypertension, CHA_2_DS_2_‐VASc score, history of atrial fibrillation, history of myocardial infarction, prior cardiovascular intervention, and preoperative systolic anterior motion status.

LA strain was the strongest four‐chamber strain marker associated with AF during follow‐up. After adjustment for age, sex, diabetes mellitus, hypertension, CHA_2_DS_2_‐VASc score, history of AF, history of myocardial infarction, prior cardiovascular intervention, and preoperative SAM status, higher LA CS was associated with lower odds of AF (adjusted OR 0.92 per 1% increase; 95% CI, 0.85–0.99; *p* = 0.018). Higher LA reservoir strain was also associated with lower odds of AF (adjusted OR 0.95 per 1% increase; 95% CI, 0.91–0.99; *p* = 0.035). RV free‐wall strain was associated with AF in unadjusted analysis but not after adjustment (adjusted OR 0.95; 95% CI, 0.90–1.01; *p* = 0.110;Table 4). No adjusted four‐chamber strain parameter was independently associated with HF hospitalization or ventricular tachycardia (Tables S4 and S5). Patients with preoperative AF had worse atrial mechanics than those without preoperative AF, including lower LA reservoir strain (18.2% vs. 26.6%; *p* < 0.001), lower LA CS (6.2% vs. 11.3%; *p* < 0.001), and lower RA reservoir strain (33.0% vs. 39.0%; *p* = 0.020), whereas baseline LV‐GLS, RV‐GLS, and RV free‐wall strain did not differ significantly (Table S6). In a sensitivity analysis excluding patients with preoperative AF, LA CS and LA reservoir strain remained independently associated with new‐onset AF (adjusted OR 0.88; *p* = 0.005 and adjusted OR 0.93; *p* = 0.016, respectively), whereas LV, RV, and RA strain parameters were not significant predictors (Table S7). When patients were grouped by preoperative NYHA functional class, LA reservoir strain and LA CS did not differ significantly between NYHA class I/II and class III/IV (25.5% vs. 24.7%; *p* = 0.491 and 11.7% vs. 9.7%; *p* = 0.258, respectively; Table S8).

**TABLE 4:** Association between baseline four‐chamber strain and atrial fibrillation during follow‐up.

Strain parameter	N	Unadjusted OR (95% CI)	*p* value	Adjusted OR (95% CI)	*p* value
LA contractile strain, %	164	0.90 (0.85–0.96)	0.002	0.92 (0.85–0.99)	0.018
LA reservoir strain, %	169	0.94 (0.91–0.98)	0.002	0.95 (0.91–0.99)	0.035
LV global longitudinal strain, %	169	0.98 (0.90–1.06)	0.560	0.98 (0.89–1.08)	0.690
RA contractile strain, %	151	0.99 (0.94–1.04)	0.570	0.98 (0.92–1.04)	0.460
RA reservoir strain, %	161	1.00 (0.97–1.03)	0.920	1.01 (0.98–1.05)	0.460
RV free‐wall longitudinal strain, %	168	0.95 (0.90–0.99)	0.041	0.95 (0.90–1.01)	0.110
RV global longitudinal strain, %	168	0.96 (0.90–1.03)	0.270	0.97 (0.90–1.05)	0.480

*Note*: ORs are reported per 1% increase in strain. Multivariable models were adjusted for age, sex, diabetes mellitus, hypertension, CHA_2_DS_2_‐VASc score, history of atrial fibrillation, history of myocardial infarction, prior cardiovascular intervention, and preoperative systolic anterior motion status.

### 3.6 Receiver Operating Characteristic Analyses

**FIGURE 2:**
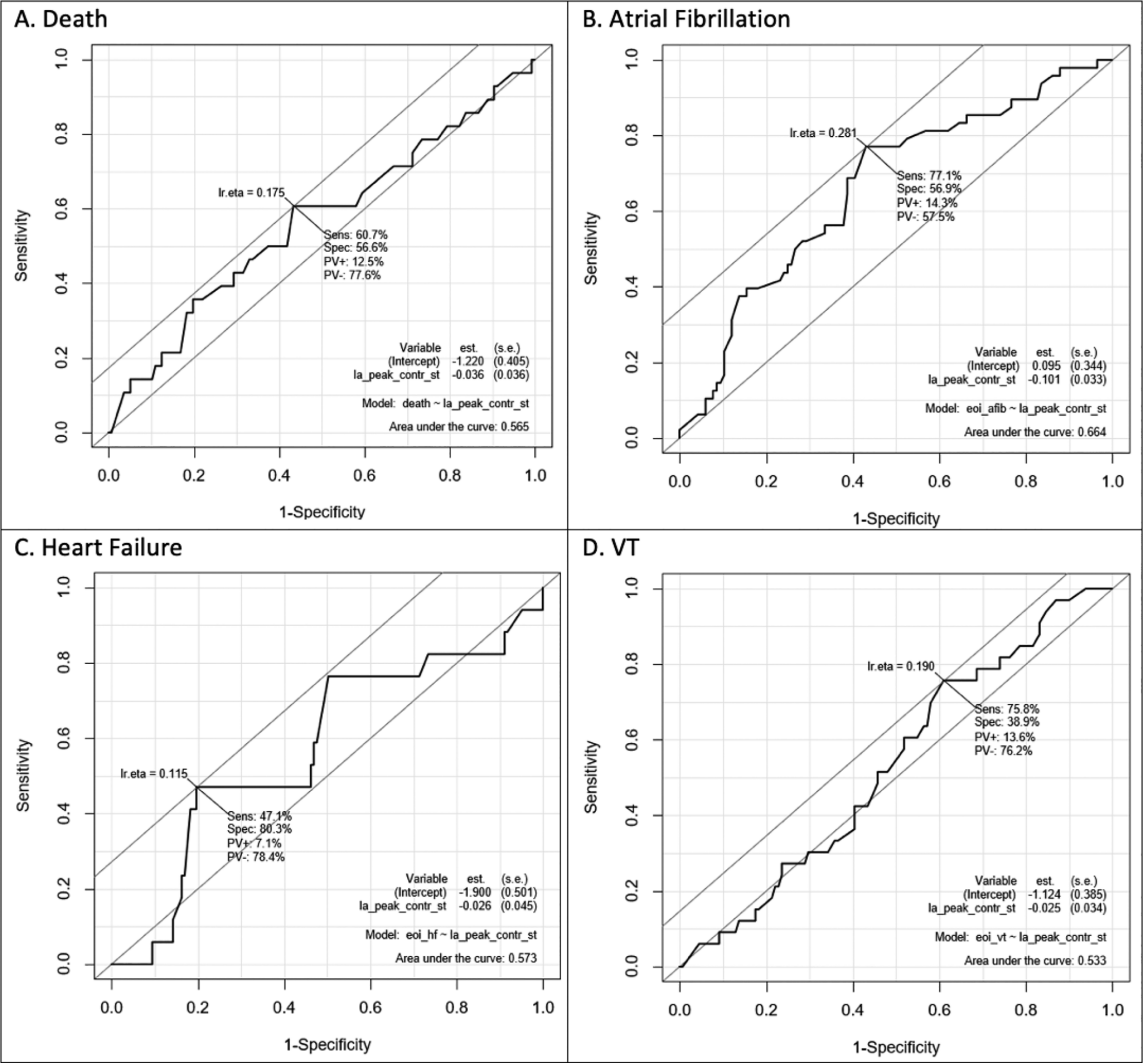
ROC curves to determine the cutoff values of left atrial peak contractile strain.

ROC analyses demonstrated modest discrimination for most outcomes. LV‐GLS showed limited discrimination across endpoints, and RV free‐wall strain showed only modest discrimination for AF. In contrast, LA reservoir strain and LA CS showed the strongest discrimination for AF among the strain measures, with AUC values of 0.668 and 0.664, respectively. These findings were consistent with the multivariable models and support LA mechanics as the strain domain most closely associated with AF during follow‐up (Figure 2 and Figures S1–S4).

## 4 Discussion

This study documents 4‐chamber remodeling that occurs following surgical myectomy for HOCM using speckle‐tracking to measure changes that occur in myocardial strain in each cardiac chamber. In our analysis of 187 consecutive patients undergoing septal myectomy for HOCM, the three most important findings were: 1) that remodeling after myectomy is chamber‐specific, 2) preoperative LA strain best correlates with postoperative AF risk, and 3) no single preoperative global strain value or its postoperative changes independently predicts sudden death.

Successful reduction of LVOT gradients with an associated reduction in LA size does not necessarily convey normal remodeling across all chambers of the heart. Rather, the heart's four chambers remodel independently depending on local myocardial pathology, postoperative loading conditions, and the physiologic consequences of open cardiac surgery. In our study, LA pump strain improved significantly at 1 year, whereas LA reservoir strain did not significantly change. The stability of the LA reservoir strain is consistent with studies suggesting that this parameter may be more dependent on an underlying atrial myopathy than on the degree of LVOT obstruction [14]. While LA reservoir strain remains the primary measure of atrial compliance and fibrosis, it may not be completely reversible following surgery [19, 20]. The improvement in LA pump strain unassociated with a change in LA reservoir strain post‐myectomy is consistent with the concept that relief of LVOT obstruction primarily reduces the afterload on the left heart, facilitating LA emptying during atrial contraction and explains the LA booster pump improvement seen in our study and in other studies [4, 21]. Chronic elevation in LV filling pressures, mitral regurgitation related to systolic anterior motion, and atrial myopathy in HOCM patients contribute to LA dilation and mechanical dysfunction, which in turn predisposes to AF [19]. The additional analysis stratified by preoperative AF status strengthens this interpretation. Indeed, LA strain is the most important determinant of new‐onset AF. In multivariable models adjusted for clinical covariates, LA reservoir strain and LA CS independently predicted AF occurrence. Thus, a 1% decrease in LA reservoir strain corresponded to an approximately 5% increase in the odds of AF, whereas a 1% decrease in LA CS corresponded to an approximately 9% increase in the odds of AF. Additional analyses stratified by preoperative AF status strengthened this interpretation: patients with preoperative AF had substantially lower LA reservoir and CS than those without preoperative AF. Moreover, after excluding patients with preoperative AF, both LA reservoir and CS remained independently associated with new‐onset AF, supporting LA mechanics as a marker of atrial myopathy and incident rhythm risk. LA strain should not be used as a stand‐alone indication for myectomy, but may complement symptoms, obstructive physiology, mitral regurgitation, exercise findings, anatomy, and response to medical therapy when considering rhythm surveillance and timing of referral. Prospective multicenter validation is needed before LA strain can be incorporated into surgical timing decisions. The mortality findings should be interpreted more cautiously. Higher LA reservoir strain was associated with lower mortality risk in unadjusted analysis (*p* = 0.015) but this association did not persist after multivariable adjustment. Similarly, LV‐GLS, RV strain, and RA strain were not independently associated with mortality in adjusted models. Therefore, these data do not support the conclusion that four‐chamber global strain independently predicts survival or sudden death after myectomy. Rather, the results suggest that LA reservoir strain may be an integrative marker of disease severity, but larger cohorts with adjudicated causes of death and time‐to‐event modeling are needed before making prognostic claims.

The lack of significant improvement in LV‐GLS after myectomy is also informative. LV‐GLS may be influenced more by the underlying HOCM myocardial substrate than by the LVOT gradient itself. In other words, myectomy relieves obstruction, but it may not reverse the fibrosis, myocyte disarray, or regional contractile heterogeneity that determine longitudinal strain [22, 23]. Therefore, persistent impairment of LV‐GLS after myectomy should not be interpreted as procedural failure. It may instead reflect the residual cardiomyopathic substrate that remains after successful relief of obstruction.

The right‐heart findings are less well described in the myectomy literature and should be interpreted cautiously. RA reservoir strain decreased, and RV free‐wall and global longitudinal strain worsened at 1 year. This does not prove that patients developed clinically meaningful RV failure, nor does it establish right‐heart strain as a prognostic marker in this postoperative cohort. Indeed, RV and RA strain parameters were not independently associated with the clinical outcomes examined. Rather, these findings show that right‐sided longitudinal deformation may change in a direction opposite to the improvement in LVOT obstruction. Several mechanisms could explain this finding. RV longitudinal mechanics are sensitive to pericardiotomy, cardiopulmonary bypass, cardioplegic arrest, postoperative septal motion, and changes in loading conditions [15, 24]. In addition, concomitant procedures were common in this cohort, including mitral valve surgery and surgical AF ablation, which may affect atrial mechanics and atrioventricular coupling [25, 26].

The practical implication is that postoperative symptoms after myectomy should not be interpreted only through the lens of LVOT gradient reduction. In some patients, persistent dyspnea or exercise limitation may reflect atrial myopathy, right‐heart deformation abnormalities, diastolic dysfunction, or residual cardiomyopathic substrate rather than recurrent obstruction. Four‐chamber strain analysis may therefore provide complementary information to conventional echocardiography, particularly when symptoms persist despite adequate relief of the LVOT gradient.

Taken together, these findings support a more nuanced view of post‐myectomy remodeling. Septal myectomy corrects the obstructive physiology of HOCM, but the postoperative heart does not remodel uniformly. LA pump strain may improve, LA reservoir strain may remain abnormal, LV‐GLS may remain largely unchanged, and right‐sided longitudinal deformation may worsen. The most clinically actionable finding from this analysis is the association between LA strain and AF during follow‐up. LA strain may therefore be useful as a quantitative marker of atrial myopathy and may help identify patients who require closer rhythm surveillance after myectomy.

### 4.1 Limitations

This study has limitations inherent in all retrospective single‐center studies. Not all patients had sufficiently clear TTE images to allow paired comparisons at 1 year, which may introduce selection and survivorship bias. Strain feasibility also varied by chamber and time point because image acquisition characteristics differed across the study period. These findings require validation in larger prospective multicenter studies.

## 5 Conclusions

In this study of 187 consecutive HOCM patients who underwent surgical septal myectomy, subsequent cardiac remodeling was chamber‐dependent and did not always follow pre‐conceived expectations. LA pump strain improved, LV‐GLS remained largely unchanged, and RA reservoir and RV longitudinal strain worsened. Decreased LA reservoir and CS were the strongest predictors of AF occurrence, suggesting that they may be useful markers of primary atrial myopathy. Surprisingly, post‐myectomy global LV and RV strain were not associated with mortality, which highlights the complexity of post‐myectomy remodeling and supports a multi‐chamber approach to postoperative imaging surveillance.

## Funding

The authors have nothing to report.

## Disclosures

Patrick M. McCarthy: Edwards Lifesciences: speaking fees and royalties; Atricure: speaking fees; Arthrex: advisory board; Genesee: royalties; Abbott: Surgical primary investigator REPAIR‐MR Trial (unpaid); advisory board. James D. Thomas: Abbott, Caption, Echo IQ, GE Healthcare, Eko Healthcare: Consulting. James L. Cox: consulting fees and meeting/travel support from Atricure and Adagio Medical; leadership roles and shareholder with Atricure, Adagio Medical, PAVmed, Lucid Diagnostics. Douglas R. Johnston: Consultant for Abbott Labs, Edwards Lifesciences, Terumo Cardiovascular, Liva Nova, Artivion, Corcym Inc., and Medtronic. Remaining authors report no disclosures.

## Ethics Statement and Consent

Approved by Northwestern University IRB (STU00012288) with written consent obtained for database participation

## Supporting information




**Supporting Information**: echo70567‐sup‐0001‐FigureS1.tif

**Supporting Information**: echo70567‐sup‐0002‐FigureS2.tif

**Supporting Information**: echo70567‐sup‐0003‐FigureS3.tif

**Supporting Information**: echo70567‐sup‐0004‐FigureS4.tif

**Supporting Information**: echo70567‐sup‐0005‐SuppMat.docx

## Data Availability

All data presented in the article and supplemental materials.
