# Supplementary figures and images for: Four‐Chamber Deformation Remodeling and Atrial Fibrillation After Septal Myectomy for Obstructive Hypertrophic Cardiomyopathy
Source: Echocardiography. 2026 Jul 28;43(8):e70567. doi: 10.1111/echo.70567 (PMC13411645; doi:10.1111/echo.70567)

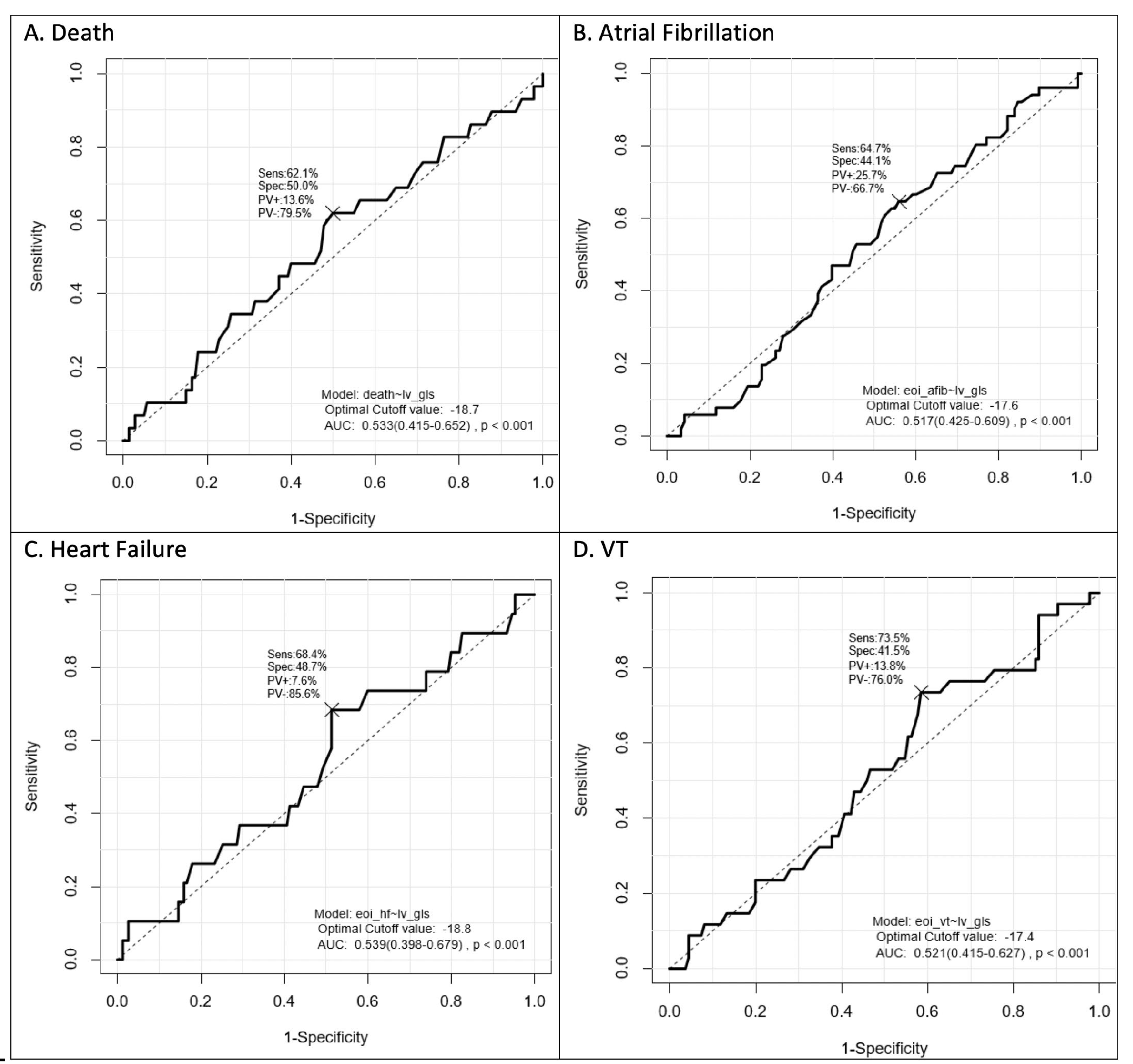

Supplement: Supplementary file 1 — Supporting Information: echo70567‐sup‐0001‐FigureS1.tif [file ECHO-43-e70567-s004.tif]

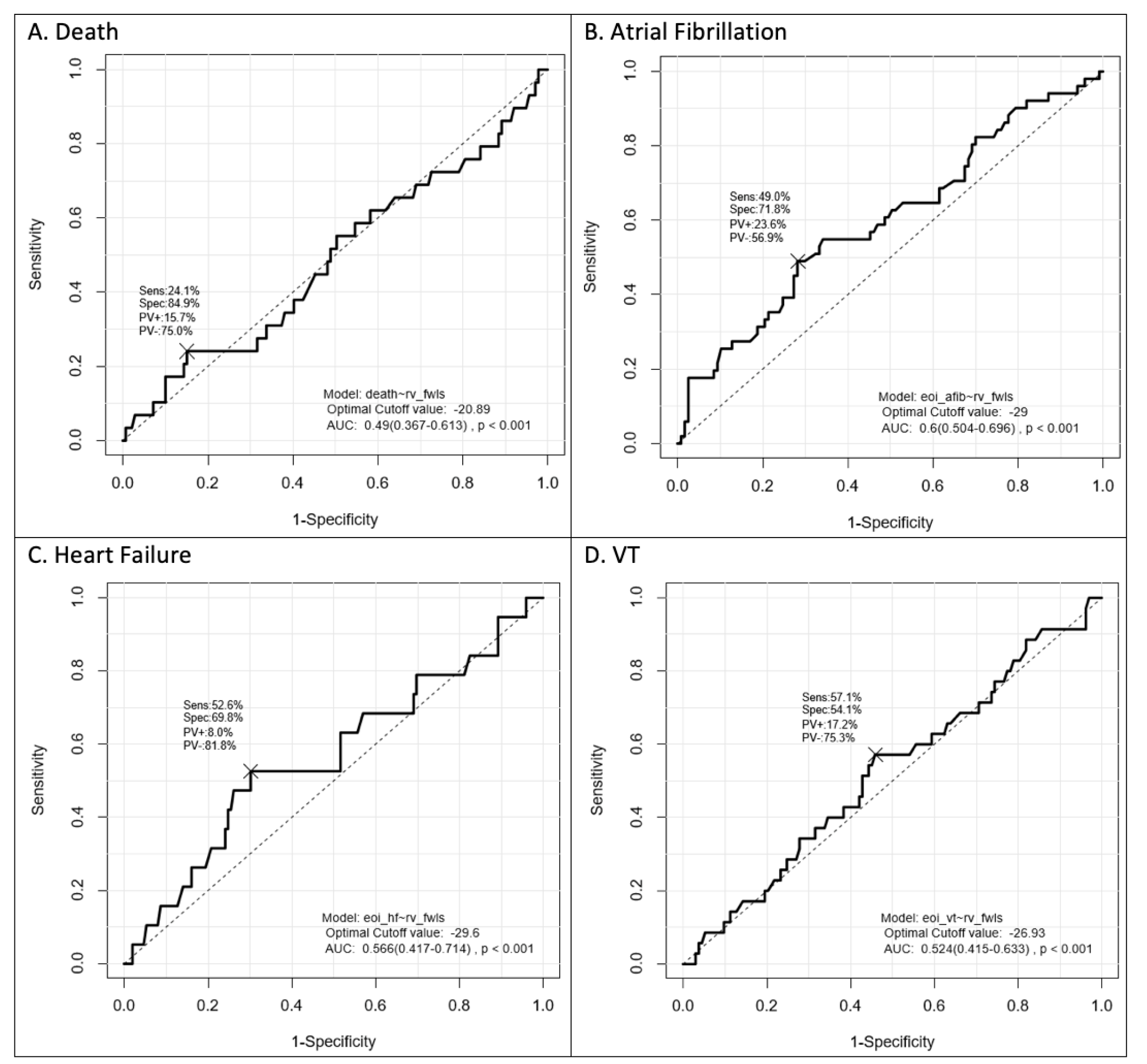

Supplement: Supplementary file 2 — Supporting Information: echo70567‐sup‐0002‐FigureS2.tif [file ECHO-43-e70567-s001.tif]

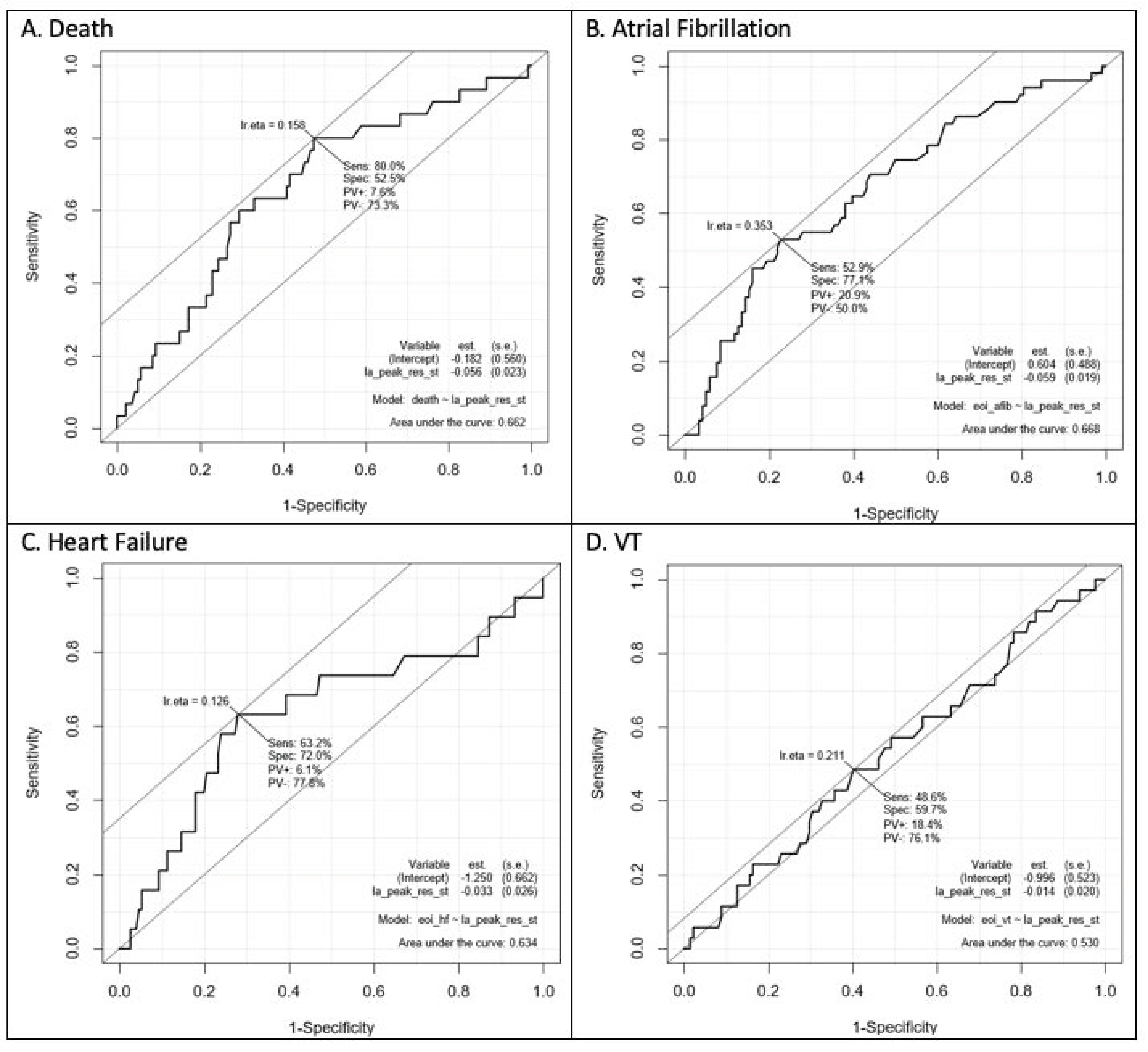

Supplement: Supplementary file 3 — Supporting Information: echo70567‐sup‐0003‐FigureS3.tif [file ECHO-43-e70567-s003.tif]

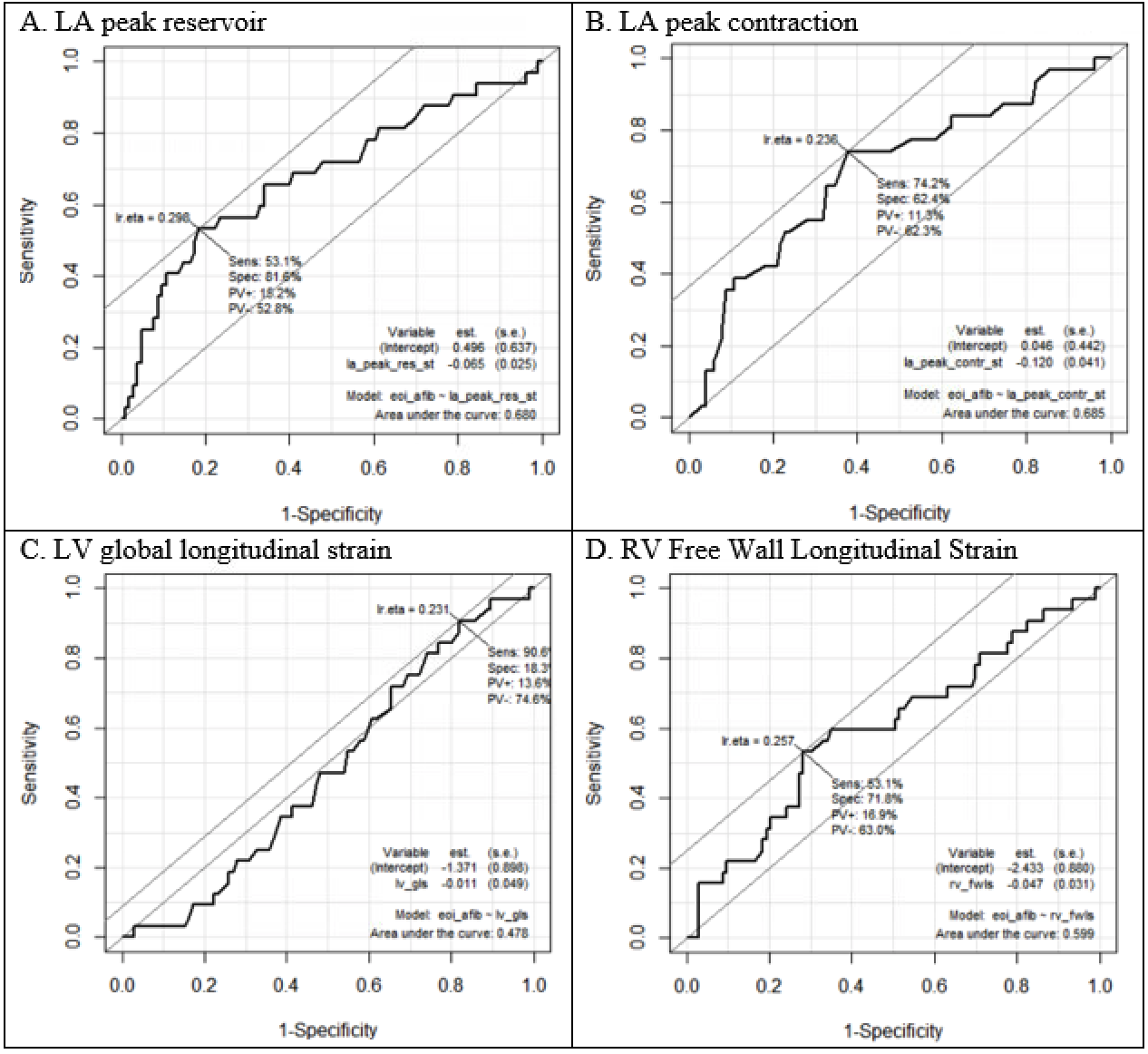

Supplement: Supplementary file 4 — Supporting Information: echo70567‐sup‐0004‐FigureS4.tif [file ECHO-43-e70567-s005.tif]
