# Supplementary material for: Four‐Chamber Deformation Remodeling and Atrial Fibrillation After Septal Myectomy for Obstructive Hypertrophic Cardiomyopathy
Source: Echocardiography. 2026 Jul 28;43(8):e70567. doi: 10.1111/echo.70567 (PMC13411645; doi:10.1111/echo.70567)
Supplement: Supplementary file 5 — Supporting Information: echo70567‐sup‐0005‐SuppMat.docx [file ECHO-43-e70567-s002.docx]

## Supplemental material:

## Supplemental Table 1. Key Postoperative Characteristics

Values are mean ± SD or n (%), unless otherwise specified

.

| Variable | Value |
| --- | --- |
| ICU stay, hours | 61.1 ± 85.6 |
| Postoperative length of stay, days | 6.0 ± 5.8 |
| Readmission within 30 days | 14 (7.8%) |
| Reoperation for bleeding | 5 (2.7%) |
| Postoperative stroke (>24 h) | 4 (2.1%) |
| Prolonged ventilation (>24 h) | 13 (7.0%) |
| Renal failure | 7 (3.7%) |
| Permanent pacemaker before discharge | 18 (9.6%) |
| Operative/30-day mortality | 5 (2.7%)* |

## Supplemental Table 2. Paired Preoperative vs 1-Year Conventional Echocardiographic Measures

*Values are mean ± SD. Δ = Post -Pre.*

| Measure | Paired N | Preoperative | 1-year post | Δ | P value |
| --- | --- | --- | --- | --- | --- |
| LVOT peak gradient, mmHg | 141 | 50.6 ± 46.7 | 10.4 ± 14.9 | −42.18 ± 46.95 | 0.001 |
| LVOT mean gradient, mmHg | 18 | 12.6 ± 23.4 | 5.0 ± 4.0 | −3.73 ± 8.71 | 0.090 |
| Interventricular septal thickness, IVSd, cm | 18 | 1.8 ± 0.4 | 1.4 ± 0.4 | −0.41 ± 0.53 | 0.004 |
| Left atrial volume, mL | 28 | 96.9 ± 32.6 | 74.2 ± 26.9 | −18.66 ± 33.39 | 0.006 |
| LV internal diameter, diastole, cm | 18 | 4.1 ± 0.6 | 4.5 ± 0.6 | +0.25 ± 0.72 | 0.160 |
| LV internal diameter, systole, cm | 18 | 2.5 ± 0.5 | 3.0 ± 0.5 | +0.51 ± 0.63 | 0.004 |
| LV ejection fraction, % | 19 | 67.0 ± 8.7 | 63.1 ± 6.2 | −2.03 ± 10.50 | 0.410 |
| RV systolic pressure, mmHg | 9 | 36.2 ± 15.9 | 28.1 ± 6.5 | −8.73 ± 9.95 | 0.030 |
| Tricuspid regurgitation peak gradient, mmHg | 9 | 29.5 ± 13.4 | 25.1 ± 8.9 | −7.72 ± 11.36 | 0.080 |

**Important change:** LVOT peak gradient should be the first row because it confirms successful relief of obstruction. It also has a much better paired N than many other conventional echo variables.

#

## Supplemental Table 3. Paired Change in Four-Chamber Strain at 1 Year

## Values are mean ± SD. Δ = 1 year - baseline.

| **Strain parameter, %** | **Paired N** | **Baseline** | **1 year** | **Δ** | **P value** |
| --- | --- | --- | --- | --- | --- |
| LA contractile strain | 71 | 10.4 ± 5.9 | 13.6 ± 7.1 | +3.01 ± 8.72 | 0.005 |
| LA reservoir strain | 75 | 25.6 ± 9.9 | 24.5 ± 11.4 | −1.82 ± 12.36 | 0.210 |
| LV global longitudinal strain | 75 | −17.9 ± 4.3 | −16.5 ± 4.5 | +0.84 ± 5.22 | 0.170 |
| RA contractile strain | 63 | 14.7 ± 6.9 | 13.7 ± 7.1 | −0.57 ± 8.95 | 0.620 |
| RA reservoir strain | 66 | 36.0 ± 10.6 | 28.0 ± 11.6 | −7.30 ± 14.09 | 0.001 |
| RV free-wall longitudinal strain | 75 | −26.3 ± 7.0 | −21.1 ± 6.8 | +4.36 ± 9.07 | 0.001 |
| RV global longitudinal strain | 72 | −21.3 ± 5.1 | −17.3 ± 5.5 | +3.42 ± 6.60 | 0.001 |

ORs are reported per 1% increase in strain. Multivariable models were adjusted for age, sex, diabetes mellitus, hypertension, CHA₂DS₂-VASc score, history of atrial fibrillation, history of myocardial infarction, prior cardiovascular intervention, and preoperative systolic anterior motion status.

## Supplemental Table 4. Association Between Four-Chamber Strain and Heart Failure Admission

| **Strain parameter** | **N** | **Unadjusted OR (95% CI)** | **P value** | **Adjusted OR (95% CI)** | **P value** |
| --- | --- | --- | --- | --- | --- |
| LA contractile strain, % | 164 | 0.97 (0.89–1.06) | 0.56 | 0.98 (0.89–1.08) | 0.74 |
| LA reservoir strain, % | 169 | 0.97 (0.92–1.02) | 0.21 | 0.99 (0.93–1.05) | 0.65 |
| LV global longitudinal strain, % | 169 | 1.02 (0.92–1.13) | 0.70 | 1.01 (0.89–1.15) | 0.82 |
| RA contractile strain, % | 151 | 0.98 (0.91–1.06) | 0.63 | 0.98 (0.90–1.08) | 0.73 |
| RA reservoir strain, % | 161 | 0.98 (0.93–1.02) | 0.27 | 0.99 (0.94–1.04) | 0.62 |
| RV free-wall longitudinal strain, % | 168 | 0.97 (0.90–1.04) | 0.42 | 0.97 (0.89–1.05) | 0.43 |
| RV global longitudinal strain, % | 168 | 0.98 (0.89–1.08) | 0.71 | 0.99 (0.89–1.10) | 0.88 |

## Supplemental Table 5. Association Between Four-Chamber Strain and Ventricular Tachycardia

| **Strain parameter** | **N** | **Unadjusted OR (95% CI)** | **P value** | **Adjusted OR (95% CI)** | **P value** |
| --- | --- | --- | --- | --- | --- |
| LA contractile strain, % | 164 | 0.98 (0.91–1.04) | 0.46 | 1.01 (0.94–1.09) | 0.75 |
| LA reservoir strain, % | 169 | 0.99 (0.95–1.02) | 0.48 | 1.00 (0.96–1.05) | 0.90 |
| LV global longitudinal strain, % | 169 | 0.97 (0.88–1.08) | 0.61 | 0.99 (0.89–1.10) | 0.80 |
| RA contractile strain, % | 151 | 0.97 (0.91–1.03) | 0.26 | 0.97 (0.91–1.04) | 0.39 |
| RA reservoir strain, % | 161 | 0.99 (0.96–1.03) | 0.70 | 1.00 (0.96–1.04) | 0.87 |
| RV free-wall longitudinal strain, % | 168 | 0.99 (0.94–1.05) | 0.74 | 1.00 (0.95–1.07) | 0.89 |
| RV global longitudinal strain, % | 168 | 1.00 (0.93–1.07) | 0.97 | 1.01 (0.94–1.09) | 0.78 |

ORs are reported per 1% increase in strain. Multivariable models were adjusted for age, sex, diabetes mellitus, hypertension, CHA₂DS₂-VASc score, history of atrial fibrillation, history of myocardial infarction, prior cardiovascular intervention, and preoperative systolic anterior motion status.

## Supplemental Table 6. Four-Chamber Strain Characteristics Stratified by Preoperative Atrial Fibrillation Status

## Values are median (Q1, Q3) unless otherwise specified.

| **Parameter** | **No Preop AF (N=151)** | **Preop AF (n=36)** | **P value** |
| --- | --- | --- | --- |
| **Preoperative four-chamber strain** |  |  |  |
| LV global longitudinal strain, % | -18.6 (-20.2, -16.5) | -18.4 (-20.7, -16.7) | 0.606 |
| RV global longitudinal strain, % | -21.3 (-24.7, -17.7) | -20.7 (-26.3, -17.7) | 0.718 |
| RV free-wall longitudinal strain, % | -26.8 (-30.7, -22.0) | -26.0 (-33.4, -23.3) | 0.742 |
| LA Volume Index, mL/m² | 45.7 (38.0, 57.3) | 57.2 (46.6, 62.6) | 0.089 |
| LA reservoir strain, % | 26.6 (20.3, 32.2) | 18.2 (12.5, 27.1) | <.001 |
| LA contractile strain, % | 11.3 (6.7, 15.0) | 6.2 (3.4, 9.2) | <.001 |
| RA reservoir strain, % | 39.0 (31.0, 43.8) | 33.0 (26.7, 40.2) | 0.020 |
| RA contractile strain, % | 14.7 (10.0, 19.3) | 13.8 (9.5, 17.1) | 0.208 |
| **One-year follow-up four-chamber strain** |  |  |  |
| LV global longitudinal strain, % | -17.3 (-20.5, -14.7) | -12.8 (-17.9, -10.7) | 0.019 |
| RV global longitudinal strain, % | -17.1 (-21.5, -14.3) | -17.2 (-19.5, -11.1) | 0.297 |
| RV free-wall longitudinal strain, % | -21.8 (-26.4, -17.3) | -21.7 (-23.9-, -16.1) | 0.517 |
| LA reservoir strain, % | 25.5 (20.0, 35.3) | 11.9 (7.8, 17.5) | <.001 |
| LA contractile strain, % | 13.9 (10.3, 19.4) | 6.7 (5.1, 8.9) | 0.001 |
| RA reservoir strain, % | 30.0 (19.6, 36.2) | 21.3 (7.3, 30.2) | 0.061 |
| RA contractile strain, % | 13.5 (9.3, 19.2) | 10.5 (5.5, 18.7) | 0.308 |

*AF = atrial fibrillation; LV = left ventricular; RV = right ventricular; LA = left atrial; RA = right atrial; LVOT = left ventricular outflow tract; TAPSE = tricuspid annular plane systolic excursion. Ventricular strain values are expressed as negative percentages,*

**Supplementary Table 7. Association Between Baseline Four-Chamber Strain and New Onset Atrial Fibrillation During Follow-up**.

| **Strain parameter** | **N** | **Unadjusted OR (95% CI)** | **P value** | **Adjusted OR (95% CI)** | **P value** |
| --- | --- | --- | --- | --- | --- |
| LA contractile strain, % | 132 | 0.89 (0.82, 0.96) | 0.004 | 0.88 (0.81, 0.96) | 0.005 |
| LA reservoir strain, % | 135 | 0.94 (0.89, 0.98) | 0.009 | 0.93 (0.88, 0.99) | 0.016 |
| LV global longitudinal strain, % | 136 | 0.99 (0.90, 1.09) | 0.830 | 1.00 (0.90, 1.11) | 0.990 |
| RA contractile strain, % | 121 | 0.98 (0.92, 1.04) | 0.460 | 0.97 (0.90, 1.03) | 0.320 |
| RA reservoir strain, % | 128 | 1.01 (0.97, 1.05) | 0.610 | 1.01 (0.96, 1.06) | 0.740 |
| RV free-wall longitudinal strain, % | 135 | 0.95 (0.90, 1.01) | 0.130 | 0.95 (0.89, 1.02) | 0.180 |
| RV global longitudinal strain, % | 135 | 0.98 (0.90, 1.06) | 0.550 | 0.98 (0.90, 1.08) | 0.690 |

*ORs are reported per 1% increase in strain. Multivariable models were adjusted for age, sex, diabetes mellitus, hypertension, CHA₂DS₂-VASc score, history of myocardial infarction, prior cardiovascular intervention, and preoperative systolic anterior motion status.*

## Supplemental Table 8. Four-Chamber Strain Characteristics Stratified by New York Heart Association Classification

## Values are median (Q1, Q3) unless otherwise specified.

| **Parameter** | **NYHA I/II (N=78)** | **NYHA III/IV (n=82)** | **P value** |
| --- | --- | --- | --- |
| **Preoperative four-chamber strain** |  |  |  |
| LV global longitudinal strain, % | -18.3 (-20.2, -16.5) | -18.7 (-20.6, -16.8) | 0.326 |
| RV global longitudinal strain, % | -21.7 (-25.1, -18.7) | -21.0 (-26.0, -17.7) | 0.885 |
| RV free-wall longitudinal strain, % | -26.8 (-31.3, -21.7) | -26.9 (-31.9, -23.0) | 0.550 |
| LA Volume Index, mL/m² | 45.2 (38.9, 53.6) | 52.9 (42.2, 62.7) | 0.156 |
| LA reservoir strain, % | 25.5 (19.0, 31.5) | 24.7 (18.3, 32.0) | 0.491 |
| LA contractile strain, % | 11.7 (6.0, 15.0) | 9.7 (6.0, 13.5) | 0.258 |
| RA reservoir strain, % | 34.9 (29.8, 41.8) | 40.0 (30.9, 43.1) | 0.125 |
| RA contractile strain, % | 13.8 (10.0, 18.1) | 15.3 (10.0, 20.0) | 0.132 |

*NYHA = New York Heart Association; LV = left ventricular; RV = right ventricular; LA = left atrial; RA = right atrial; LVOT = left ventricular outflow tract; TAPSE = tricuspid annular plane systolic excursion. Ventricular strain values are expressed as negative percentages,*

**Supplemental Figure 1. ROC curves to determine the cutoff values of Left ventricular global longitudinal strain**

**Supplemental Figure 2. ROC curves to determine the cutoff values of Right Ventricle - Free Wall Longitudinal Strain**

**Supplemental Figure 3. ROC curves to determine the cutoff values of left atrial peak reservoir strain**

**Supplemental Figure 4. ROC curves for new onset atrial fibrillation**
